# Supplementary material for: The Aging Epigenome: Integrative Analyses Reveal Functional Overlap with Alzheimer’s Disease
Source: medRxiv. 2025 Jun 9:2025.06.08.25329218. Preprint. [Version 1] doi: 10.1101/2025.06.08.25329218 (PMC12204410; doi:10.1101/2025.06.08.25329218)
Supplement: Supplement 1 [file media-1.pdf]

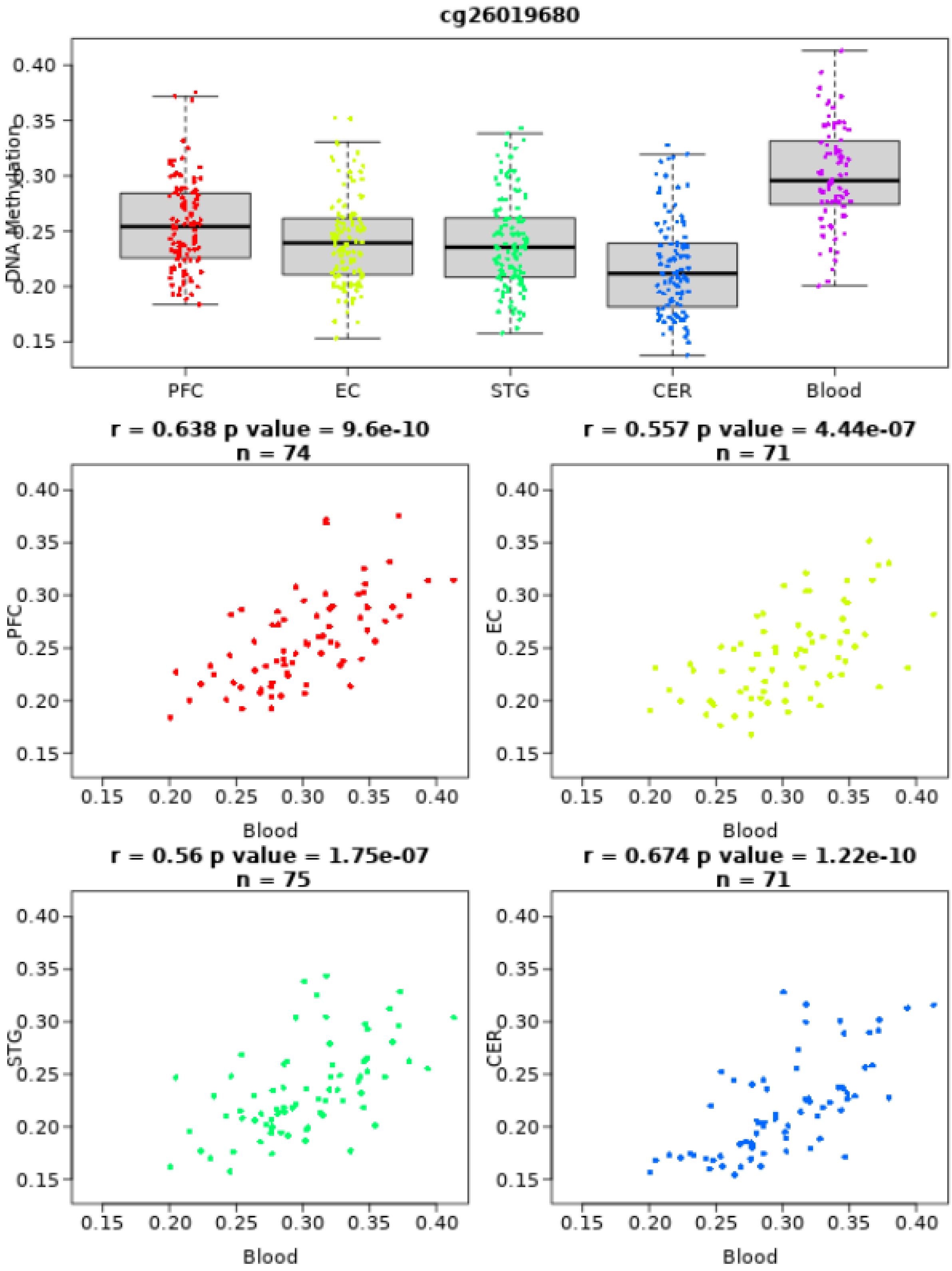

Supplementary Figure 1 Brain-blood correlations for DNA methylation levels at cg26019680 located in promoter region of the PODXL2 gene. These figures were obtained using the Blood Brain DNA Methylation Comparison Tool (https://epigenetics.essex.ac.uk/bloodbrain/?probenameg=cg26019680). **Abbreviations** PFC: Prefrontal Cortex, EC: Entorhinal Cortex, STG: Superior Temporal Gyrus, CER: Cerebellum
